# Chitosan/Hesperidin Nanoparticles for Sufficient, Compatible, Antioxidant, and Antitumor Drug Delivery Systems

**DOI:** 10.3390/ph17080999

**Published:** 2024-07-29

**Authors:** May Almukainzi, Thanaa A. El-Masry, Enas I. El Zahaby, Maysa M. F. El-Nagar

**Affiliations:** 1 Department of Pharmaceutical Sciences, College of Pharmacy, Princess Nourah bint Abdulrahman University, P.O. Box 84428, Riyadh 11671, Saudi Arabia; mkalmukainizi@pnu.edu.sa; 2 Department of Pharmacology and Toxicology, Faculty of Pharmacy, Tanta University, Tanta 31527, Egypt; thanaa.elmasri@pharm.tanta.edu.eg; 3 Department of Pharmaceutics, Faculty of Pharmacy, Delta University for Science and Technology, Gamasa 35712, Egypt; enas.elzahabi@deltauniv.edu.eg

**Keywords:** antioxidant, antitumor, chitosan, hesperidin, chitosan/hesperidin nanoformulation

## Abstract

One flavonoid glycoside with demonstrated therapeutic potential for several illnesses, including cancer, is hesperidin. However, because of its limited bioavailability and solubility, it is only marginally absorbed, necessitating a delivery mechanism to reach the intended therapeutic target. Additionally, the cytoskeleton of crustaceans yields chitosan, a naturally occurring biopolymer with mucoadhesive properties that has been used to improve the absorption of advantageous chemical substances like flavonoids. Chitosan/hesperidin nanoparticles (Hes-Nanoparticles) were made using the ion gelation technique. The synthesis of Hes-Nanoparticles was confirmed by several characterization methods, including the swelling test, zeta potential, particle size, FTIR, XRD, TEM, and SEM. DPPH and ABTS were used to demonstrate radical scavenging activity in antioxidant assays of chitosan, hesperidin, and the synthesized Hes-Nanoparticles. In addition, by a viability assay against MDA-MB-231, the anticancer efficacies of chitosan, hesperidin, and the synthesized Hes-Nanoparticles were assessed. Furthermore, annexin-V/PI double staining and the cycle of cell analysis were determined by flow cytometry. The results displayed that Hes-Nanoparticles have higher antioxidant activity than chitosan and hesperidin alone. Also, it has been demonstrated that Hes-Nanoparticles are more effective in early cell cycle arrest, suppressing the viability of cancer cells, and increasing cell apoptosis than chitosan and hesperidin alone. In conclusion, Hes-Nanoparticles demonstrated more antioxidant and antitumor activities than chitosan and hesperidin alone. Moreover, it has been established that Hes-Nanoparticles, in a highly soluble form, increase activity in contrast to the poorly soluble form of hesperidin alone.

## 1. Introduction

Breast cancer is the top cause of mortality for women worldwide and the second most common disease in terms of diagnosis globally [1]. The condition is deadly, and the list of risk factors for breast cancer appears to be growing daily. Numerous endogenous and exogenous variables have the potential to exacerbate the pathophysiology of breast cancer [2]. Treating patients with breast cancer is becoming increasingly difficult due to several factors, such as adverse effects from traditional treatments, including radiation and chemotherapy [3]. Multidrug resistance (MDR) is one of the most dangerous issues associated with traditional therapy [4].

Natural products with a wide range of origins may have the ability to activate several physiological pathways, which may be advantageous for the treatment of chronic illnesses such as cancer [5,6]. For many years, it has been difficult to properly treat cancer; as a result, using a variety of treatment approaches is now required. Compounds originating from plants are being investigated to help answer this difficult riddle. 

Many studies have been conducted to develop natural substances, particularly phytochemicals, as cancer treatments [7]. According to recent research, natural chemicals derived from food sources may be able to target certain breast-cancer-related pathways, which may have a protective effect against cancers and be crucial in treating breast cancer [6,8]. A class of polyphenolic substances known as flavonoids are produced by plants as secondary metabolites. Numerous fruits, vegetables, and other food crops contain flavonoids. In addition to other bioactivities (such as anti-inflammation and anti-aging), they have positive biochemical effects on several disorders (such as cancer, atherosclerosis, and cardiovascular disease) [8,9]. 

Hesperidin (Hes) is a glycoside flavanone. Numerous research works have shown hesperidin’s pharmacokinetics, bioavailability, and absorption characteristics. It is commonly recognized that hesperidin is a strong natural antioxidant that can lower oxidative stress [10,11,12]. Numerous pharmacological characteristics, such as anti-inflammatory, antibacterial, anticarcinogenic, antithrombotic, and antiviral activity, have been reported for hesperidin [13,14]. Hes is a promising chemical, but it is rapidly excreted and has limited bioavailability due to its strong plasma protein binding [11]. 

Despite its important biological functions, hesperidin is often fragile and prone to breakdown, and it can interact negatively with dietary components such as proteins. Hesperidin has a low partition coefficient (log Poctanol/water = 0.30) and low water solubility (5.92 ± 0.49 μg/mL at 25 °C), resulting in low bioavailability [15].

Therefore, an inventive Hes formulation is necessary. Various research teams are now working to increase the bioavailability of flavonoids using techniques like cyclodextrin complexes, phospholipid complexes, and nanoformulation, among other modern drug delivery strategies [16]. Nanoparticles exhibit biomimetic properties because of their high surface ratio [17]. The surface, solubility, size, shape, bioavailability, and biodistribution of nanoparticles are their main advantages in drug administration.

Chitosan (Cs) is a naturally occurring biopolymer with mucoadhesive characteristics; it is produced from the cytoskeleton of crustaceans [18] and has been applied to augment the absorption of advantageous chemical substances like flavonoids [19,20]. For instance, it has been proven that combining tea polyphenols with chitosan nanoparticles will increase the bioavailability and absorption of the phenols [21]. The most important functional component of the biological activity of Cs is its primary amine group. This main amino group is the fundamental functional group of chitosan that allows for interactions with other molecules. Additionally, this polymer exhibits higher levels of hydrogen bond interactions at pH 5 compared to acidic pH solutions. This is explained by the greater built chain at pH 5 and the reduced protonation of amine groups. Chitosan is solvable by primary amine protonation in aqueous acidic environments. On the other hand, chitin has a sufficient amount of acetylated residues to stop polymers from dissolving in acidic aqueous solutions.

Because of their distinct biological characteristics, which include antioxidant, anti-allergic, anti-inflammatory, anti-coagulant, anti-cancer, anti-bacterial, anti-human immunodeficiency virus, anti-hypertensive, anti-Alzheimer’s, anti-diabetic, and anti-obesity effects, it is therefore believed that chitosan and hesperidin together may have a synergistic effect [22,23].

Although several studies have manufactured chitosan-loaded nanoparticles, the ion gelation method is considered the simplest and cheapest approach. Several factors affect the particle size and entrapment efficiency of nanoparticles. Our study aimed to investigate two factors: the molecular weight of chitosan and the effect of temperature (2 and 25 °C). The particle size of the nanoparticles used was considerably small and can be compared with the particle size obtained by a previous study that utilized the emulsification and evaporation method [24]. Another study employed a solvent evaporation method, in which expensive polymers were utilized, such as PLGA and soy phosphatidylcholine [25]. The biological effects of chitosan as a natural biodegradable with antioxidant and anti-inflammatory characteristics, and being the only biopolymer with a positive charge, make it an appropriate choice to achieve the study’s objective successfully (via a slight modification of experimental conditions, i.e., using low-temperature, as illustrated by the factorial design). Consequently, Hes-Nanoparticles were more effective in early cell cycle arrest, suppressing the viability of cancer cells, and increasing cell apoptosis than chitosan and hesperidin alone. The results of this study are consistent with a previous experiment utilizing hesperetin for the treatment of colorectal cancer [25]. 

Chitosan/hesperidin nanoparticles (Hes-Nanoparticles) were prepared by the ionotropic gelation technique. This is a simple, versatile method for producing high-encapsulation-efficiency micro- and nanoparticles for medicinal and biological purposes. Many drugs have been successfully encapsulated using this technique [10]. When two biopolymers with opposing charges interact in an aqueous colloidal medium, a solution splits into two liquid phases, a phenomenon known as complex coacervation [11].

Therefore, the purpose of this investigation was to evaluate the antioxidant and antitumor activities of chitosan/hesperidin nanoparticles prepared by the ionic gelation technique in comparison with each used alone.

## 2. Results 

### 2.1. Optimization of Hesperidin Nanoparticles

**Figure 1:**
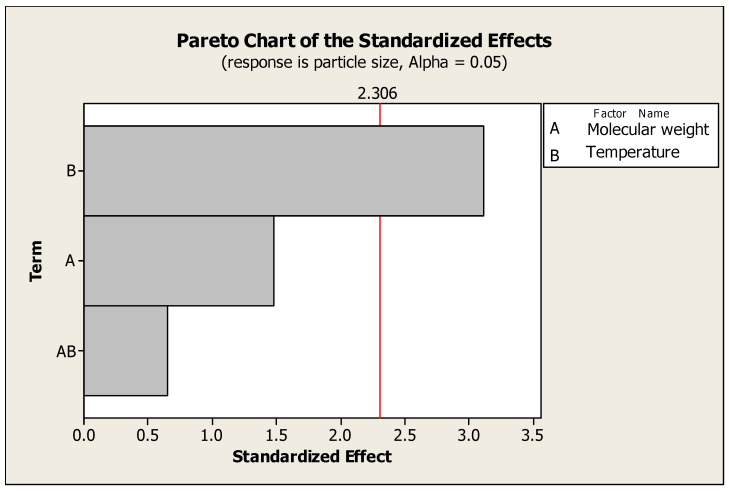
Pareto chart of the effects of temperature and chitosan’s molecular weight on zeta potential.

**Figure 2:**
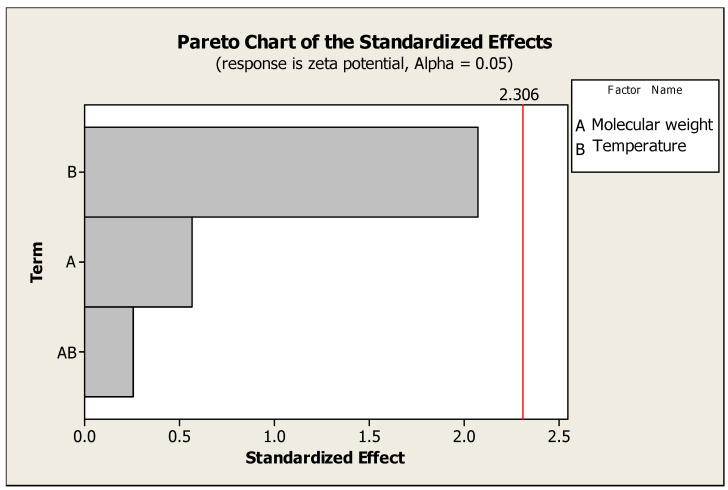
Pareto chart of the effects of temperature and chitosan’s molecular weight on particle size.

Formulation optimization was performed with the aid of Minitab 16 statistical software, based on studying two factors with two-level designs (Table 1). The first factor was chitosan’s molecular weight (100 and 300 KD) and the second was temperature (2 and 25 °C) (Table 2). The results demonstrated the significant effect of temperature on particle size; on the other hand, polymer molecular weight did not have a significant effect on the previous response (Figure 1 and Figure 2). The selected formula for Hes-Nanoparticles comprised low-molecular-weight chitosan at 2 °C.

Zeta potential (mV) = 32.5488 + (−0.0055572) Molecular weight + (−0.283551) Temperature + (−0.00046304) Molecular weight × Temperature(1)

Particle size (nm) = 73.983 + (0.239949) Molecular weight + (5.29536) Temperature + (0.0193087) Molecular weight × Temperature(2)

### 2.2. Characterization of Hes-Nanoparticles

#### 2.2.1. Swelling Test

**Figure 3:**
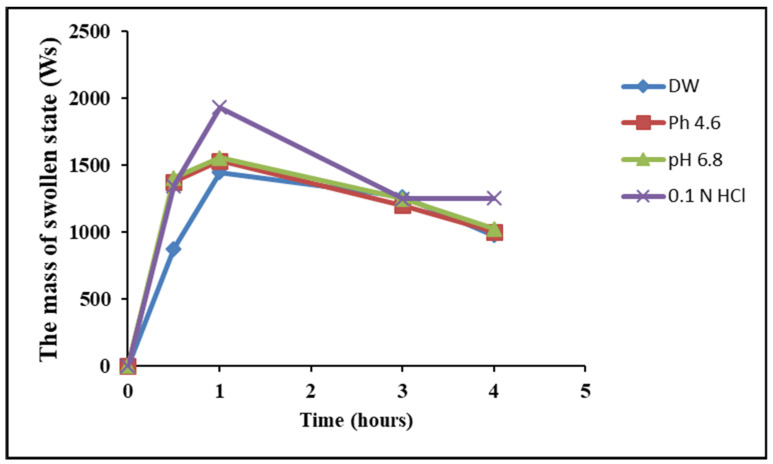
Swelling test of Hes-Nanoparticles in different media (DW, pH 4.6, 6.8, and 0.1 N HCl).

The swelling of Hes-Nanoparticles in different media was observed for 4 h (Figure 3), and the results demonstrated the highest swelling ratio in 0.1 N HCl (37.7) at pH 6.8 (30.1) after 1 h, while the sol fraction was almost the same in the different media. Figure 3 illustrates the higher swellability of Hes-Nanoparticles in 0.1 N HCl at pH 6.8 in comparison to water and pH 4.6. At 0.1 N HCl, the lowest volume swelling factor (0.42) was found (Table 3). The swelling test in 0.1 N HCl illustrated that the swelling ratio was 5.494 for Hes-Nanoparticles and zero for Hes after 72 h.

#### 2.2.2. Percentage Yield and Loading Capacity

The yield as a percentage was 91.51 ± 2.7%. The entrapment efficiency (EE) was 85.92 ± 1.9%, and the loading capacity (LC) was 30.69 ± 0.66% (Table 4).

#### 2.2.3. Particle Size and Zeta Potential Analysis

**Figure 4:**
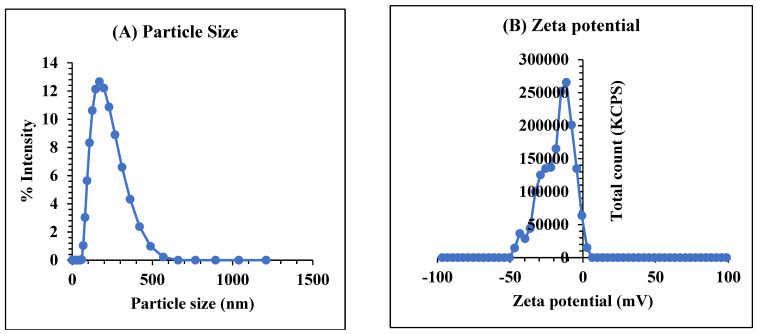
Particle size (**A**) and zeta potential (**B**) of Hes-Nanoparticles.

The generated Hes-Nanoparticles were examined utilizing dynamic light scattering (DLS), showing a single peak with an average particle size of 184.1 ± 20.03 nm (Figure 4A). Zeta potential ranged between −20.61 and −42.75 mV, with an average of −29.07 ± 9.78 mV (Figure 4B). The average polydispersity index (PDI) was 0.233 ± 0.061 (Table 3). 

#### 2.2.4. Scanning Electron Microscopy (SEM) and Transmission Electron Microscopy (TEM)

**Figure 5:**
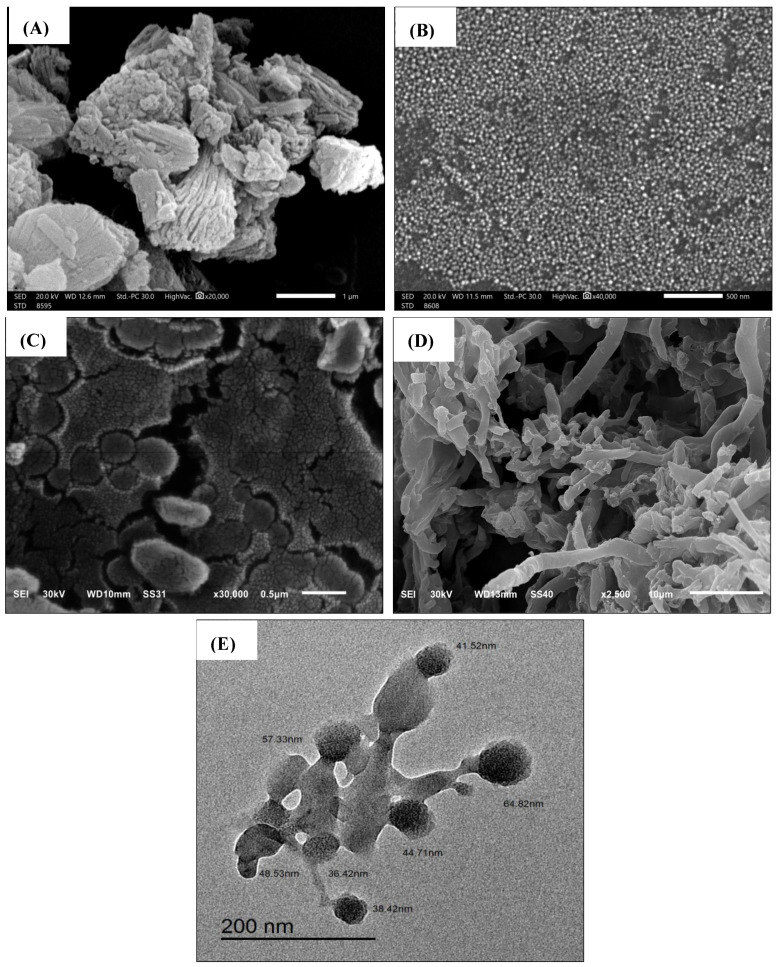
SEM images of Hes (**A**), Hes-Nanoparticles (**B**), and drug-free nanoparticles (**C**), cross-linking character of Hes-Nanoparticles (**D**), and TEM image of Nano-Hes (**E**).

The particle morphology, size, and shape of Hes and Hes-Nanoparticles were evaluated by SEM, as shown in Figure 5A. Hes particles displayed an irregular arrangement with a rough, fibrous outer surface (magnification 20,000). Nano-Hes appeared as spherical, well-separated particle aggregates (magnification 40,000) less than 40 nm in diameter (Figure 5B). Drug-free nanoparticles were characterized by regular, well-defined spherical shapes (magnification 30,000) that were quite similar to Hes-Nanoparticles (Figure 5C). Hes-Nanoparticles at lower magnification (2500) showed the cross-linking character of the spheres produced by the ionic gelation method (Figure 5D). The TEM imaging clearly illustrates the cross-linked spherical nanostructure particles with a diameter of less than 100 nm (26.84 to 66.64 nm) (Figure 5E). 

#### 2.2.5. DSC Analysis

**Figure 6:**
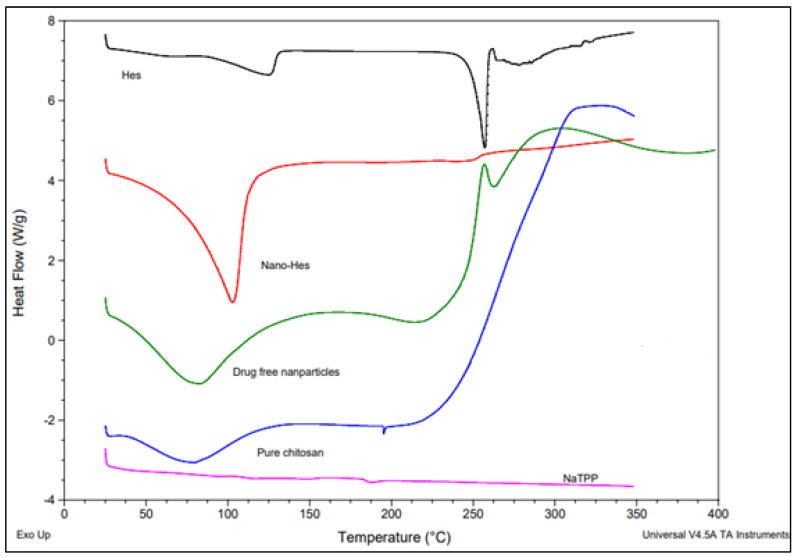
DSC of pure Hes, pure chitosan, NaTPP, Hes-Nanoparticles, and drug-free nanoparticles.

The DSC analysis was conducted for the pure components (chitosan, Hes, and NaTPP), drug-free nanoparticles, and Hes-Nanoparticles (Figure 6). The Hes DSC thermogram revealed a high endothermic peak at 257.01 °C and a minor peak at 125.22 °C, suggesting the melting of the compound and demonstrating its crystalline form [12]. An endothermic peak (the dehydration (TD) endothermic peak) [26] was visible in the pure chitosan at 80.05 °C. A broad exothermic peak between 300 and 340 °C is attributed to polymer thermal degradation (cleavage of glycoside bond and breakdown of acetyl and deacetylated units [26]). The thermogram for Nano-Hes shows the complete absence of a sharp endothermic peak (TM at 257.01), indicating the transformation into an amorphous state; however, the first peak becomes broader and shifts slightly to a lower temperature (102.97 °C, 654.1 J/g), which could be attributed to interaction with the chitosan polymer. The drug-free nanoparticle thermogram is almost identical to the DSC of pure chitosan, with two characteristic endothermic (broader peak) and exothermic peaks (plateau flat peak). The thermogram for NaTPP shows non-characteristic peaks.

#### 2.2.6. X-ray Diffraction Analysis (XRD)

**Figure 7:**
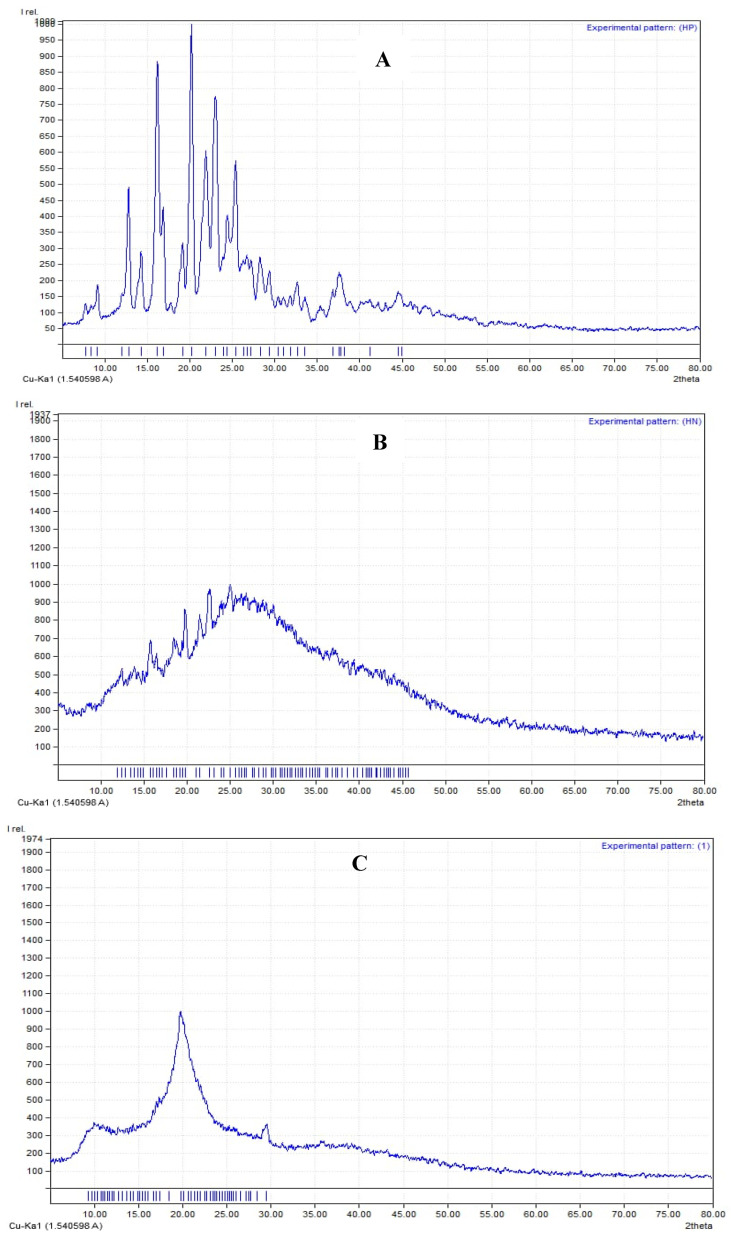
XRD of Hes (**A**), Hes-Nanoparticles (**B**), and chitosan (**C**).

XRD was performed for both Hes and Hes-Nanoparticles (X-ray powder diffractometer, GNR, Italy, at 35 KV and 25 mA). The diffractogram of Hes was examined (Figure 7A), and characteristic sharp diffraction peaks at 2θ (12.77, 14.24, 16.1, 20.18, 21.89, 22.91, and 25.43°) were identified. On the other hand, the diffractogram of Hes-Nanoparticles showed a much more diminished peak (Figure 7B).

#### 2.2.7. Fourier-Transform Infrared Spectroscopy (FTIR) Analysis

It is possible to attribute the changes in nanoparticles to the ionic interaction between TPP and the amine groups, because the bands for amine I N-H bending vibration at 1600/cm show a high protonated peak, and the amide II carbonyl stretch appears at 1670/cm, which changes to 1507/cm and 1697/cm, respectively. Further evidence of the presence or interaction of TPP is provided by the P=O peak in the cross-linked chitosan [27], which is seen at 668/cm. We therefore conclude that chitosan’s ammonium groups and the tri-polyphosphoric groups of TPP are connected.

**Figure 8:**
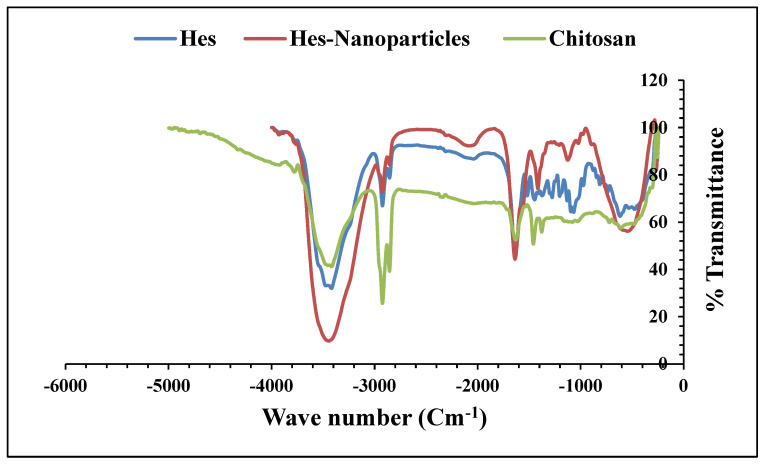
FTIR analysis of Hes, Hes-Nanoparticles, and chitosan.

Hes’s FTIR spectra (Figure 8) show a distinct strong characteristic broad peak at 3418.67/cm, which is recognized as the hydroxyl (OH) group’s stretching vibration frequency. The weak broad peak at 2923.09/cm is due to the hydroxyl stretching of alcohol. The peak at 1644.35/cm denotes the presence of functional groups with the carbonyl (C=O) prefix. The aromatic ring (stretching of -C=C-) is represented by the peak at 1517.22 cm^−1^. In response to ether linkage with -C-O-C- and -C-O stretching, nonstop peaks were seen between 1067.96/cm and −1373.43/cm. The optimized Nano-Hes (Figure 8) shows slight variations, but the distinct peaks attributed to the hydroxyl group become wider (3444.72/cm), while the peaks at 2924.23 and 1637.06/cm become less prominent. The fingerprint region (1600 to −2000/cm) shows different peaks, with a characteristic broad peak at 543.88 cm^−1^ that may result from interaction with NaTPP to produce a P=O peak. A type of interaction between the tri-phosphoric group of NaTPP and the ammonium group of chitosan was concluded [27]. Chitosan’s FTIR graph is shown below (Figure 8) 

#### 2.2.8. In Vitro Drug-Release Study

**Figure 9:**
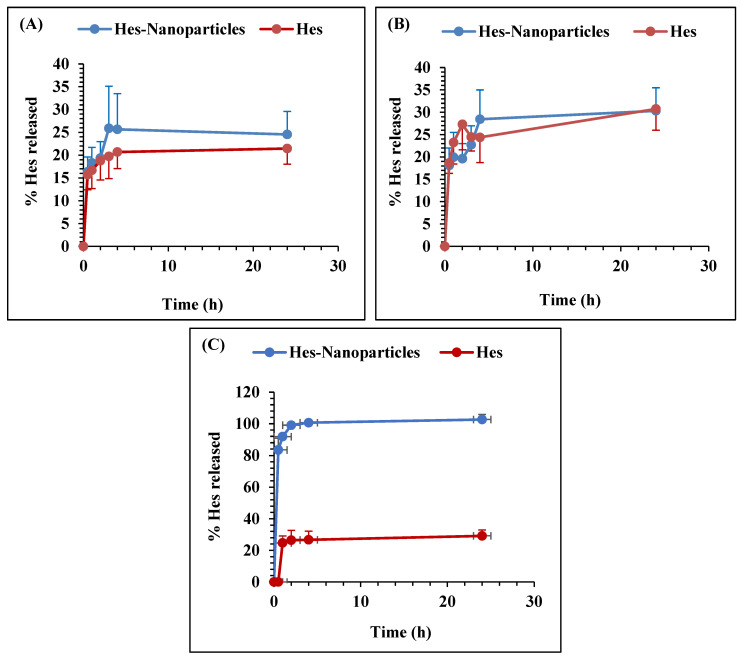
Percentage of drugs released from Hes and Hes-Nanoparticles at pH 4.6 (**A**), pH 6.8 (**B**), and in 0.1 N HCL (**C**).

The dissolution of Hes and Hes-Nanoparticles in 0.1 N HCL at pHs of 4.6 and 6.8 was performed. Figure 9 demonstrates that Hes and Nano-Hes show almost the same behavior. The percentage of drugs released at pH 4.5 was 24.5% for Hes-Nanoparticles and 21.5% for Hes. On the other hand, for pH 6.8, this was 30.3% for Nano-Hes in comparison to 30.8% for Hes. On the other hand, the dissolution in 0.1 N HCL showed a complete release of Hes from the Hes-Nanoparticles after 2 h (99.1%), while only 29.18% after 24 h was released from the Hes powder (Figure 9C).

**Figure 10:**
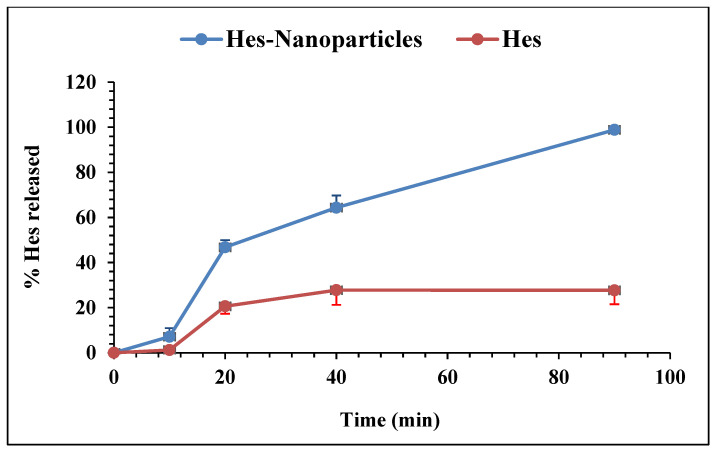
Percentage of Hes released in 0.1 N HCl.

The dissolution in 0.1 N HCl was repeated utilizing a 900 mL dissolution medium to best achieve the sink condition. The dissolution illustrated the % of drugs released from Hes and Hes-Nanoparticles (Figure 10), demonstrating the higher solubility of Hes-Nanoparticles in comparison to Hes. The proportion of the drug that had been dissolved after 20 and 90 min (Q20% and Q90%) for Hes and Hes-Nanoparticles was one of the dissolving parameters calculated. Hes’s Q20% and Q90% values were 20.63 and 27.7%, respectively, while Hes-Nanoparticles’ Q20% and 90% values were 46.8 and 98.88, respectively.

### 2.3. Antioxidant Activity Assay

#### 2.3.1. DPPH Radical Scavenging Assay

**Figure 11:**
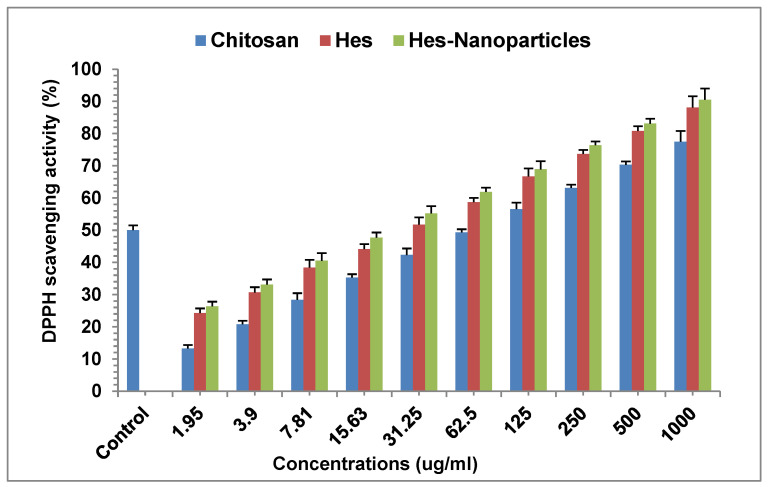
Antioxidant activity of chitosan, Hes, and Hes-Nanoparticles. Results are expressed as a mean± SD (*n* = 3). Control: ascorbic acid; Hes: hesperidin; Hes-Nanoparticles: chitosan/hesperidin nanoparticles.

The radical scavenging activity of DPPH was measured to evaluate antioxidant activity (Figure 11). In the DPPH assay, the treatments of chitosan, Hes, and Nano-Hes demonstrated concentration-dependent DPPH radical scavenging activities (Figure 11). Nano-Hes showed a higher effect than chitosan and Hes. The mean scavenging concentrations (IC_50_, expressing 50% of scavenging) were 67.44 ± 2.4, 25.4 ± 1.3, and 19.63 ± 0.9 μg/mL for chitosan, Hes, and Hes-Nanoparticles, respectively, whereas the IC_50_ of ascorbic acid as a positive control was 2.85 μg/mL (Figure 11).

#### 2.3.2. ABTS Radical Scavenging Assay

**Figure 12:**
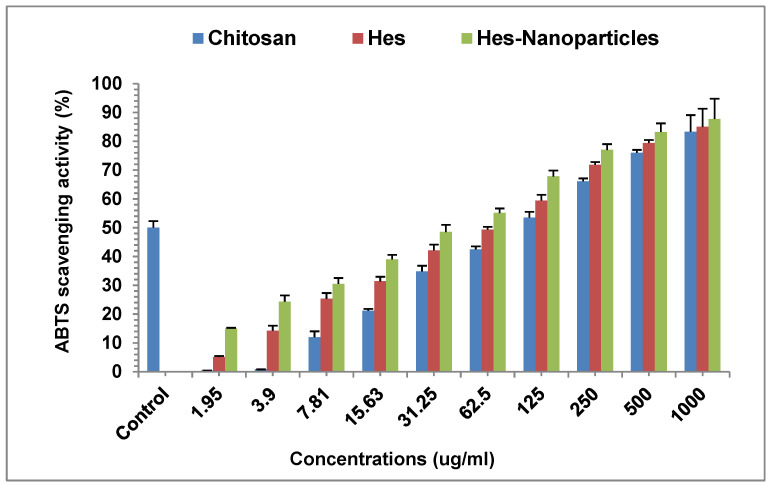
Antioxidant activity of chitosan, Hes, and Hes-Nanoparticles. Results are expressed as mean ± SD (*n* = 3). Control: gallic acid; Hes: hesperidin; Hes-Nanoparticles: chitosan/hesperidin nanoparticles.

The results of the ABTS assay were comparable to those of the DPPH assay, and the antioxidant effect of the Hes-Nanoparticles was greater than chitosan and Hes (Figure 12). The IC_50_ values of the ABTS scavenging assay were established to be 94.66 ± 5.3, 58.79 ± 2.4, and 35.41 ± 1.2 μg/mL for chitosan, Hes, and Hes-Nanoparticles, respectively; however, the IC_50_ of gallic acid as a positive control was 2.61 μg/mL (Figure 12).

### 2.4. Antitumor Activity 

#### 2.4.1. Viability Test for Chitosan, Hes, and Hes-Nanoparticles Demonstrated Anticancer Efficacy against MDA-MB-231 (Breast Cancer Cell Line)

Table 5 presents the inhibitory effects of chitosan, Hes, and Hes-Nanoparticles on a breast cancer cell line (MDA-MB-231), with IC_50_ values of 116.63 ± 2.96, 110.82 ± 5.89, and 90.83 ± 5.15 μg/mL, respectively. Chitosan was used at concentrations of 0–125 μg/mL. The lowest concentration (25 μg/mL) recorded a maximum viability of 87.44% and a lower inhibition effect of 12.65 ± 1.22%; however, the highest inhibition of 49.17 ± 2.38% was recorded with the highest concentration of free chitosan (125 μg/mL) (Table 5). 

In the same context, Hes was also used at concentrations of 0–125 μg/mL, and the lowest concentration (25 μg/mL) recorded the highest viability of 73.53 ± 1.75% with a lower inhibition effect of 26.47 ± 1.6%. The highest inhibition of 53.76 ± 1.93% was recorded with the highest amount of Hes (125 μg/mL) (Table 5).

Furthermore, Hes-Nanoparticles were also used at concentrations of 0–125 μg/mL, and the lowest concentration (25 μg/mL) recorded the highest viability of 66.69 ± 5.42% with a lower inhibition effect of 33.3 ± 2.4%. However, the highest inhibition of 63.46 ± 2.8% was recorded with the highest amount of Hes (125 μg/mL) (Table 5).

#### 2.4.2. Cell-Cycle Analysis

**Figure 13:**
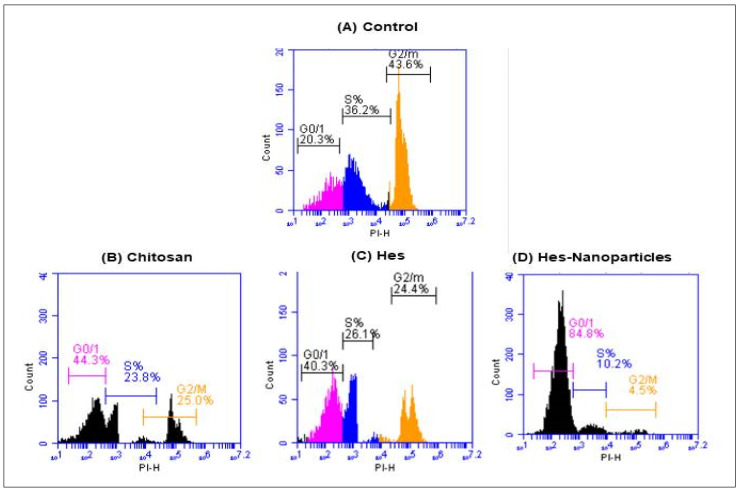
Breast cancer cell line (MDA-MB-231) cell-cycle distribution in untreated (control) (**A**) and after treatments with chitosan (**B**), Hes (**C**), and Hes-Nanoparticles (**D**) were analyzed using flow cytometric analysis based on IC_50_ concentrations detected by MTT assay. Hes: hesperidin. Hes-Nanoparticles: chitosan/hesperidin nanoparticles.

The cell-cycle analysis of chitosan, Hes, and Hes-Nanoparticles using flow cytometry is shown in Figure 13A–C, with different cell-cycle phases (G0/G1, S, and G2/M). The treatment with chitosan indicated a G2/M-phase cell-cycle arrest from 43.6% in untreated cells to 25% in treated cells. In the same context, cells treated with Hes showed a G2/M-phase cell-cycle arrest from 43.6% in untreated cells to 24.4% in treated cells. Furthermore, cells treated with Hes-Nanoparticles displayed a sharp G2/M-phase cell-cycle arrest from 43.6% in untreated cells to 4.5% in treated cells.

#### 2.4.3. Annexin V/PI Double-Staining

**Figure 14:**
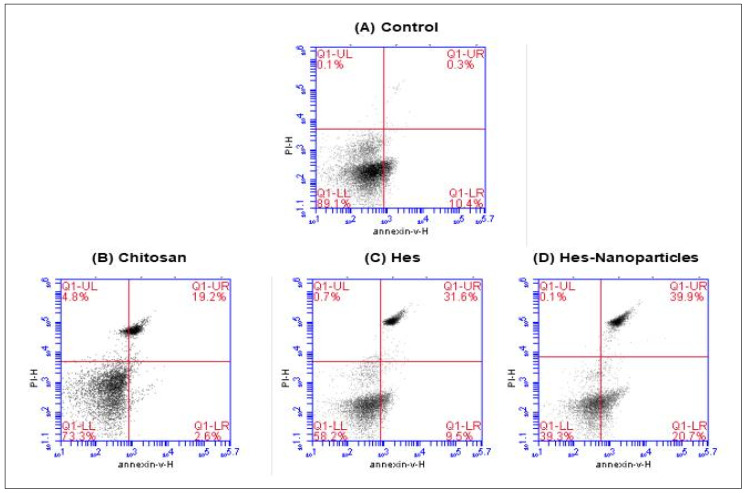
Estimation of apoptotic cell populations of breast cancer cell line (MDA-MB-231) in untreated (control) (**A**) and after treatment with chitosan (**B**), Hes (**C**), and Hes-Nanoparticles (**D**), determined by annexin V/PI double staining based on IC_50_ concentrations detected by MTT assay using flow cytometry. The lower-right quadrant shows cells in apoptosis. Hes: hesperidin. Hes-Nanoparticles: chitosan/hesperidin nanoparticles.

The apoptotic cells influenced by chitosan, Hes, and Hes-Nanoparticles were revealed by annexin-V/PI double staining, as revealed in Figure 14. The MDA-MB-231 breast cancer cell line treated with Nano-Hes showed an increase in apoptotic cells from 10.4% in untreated cells to 20.7% in treated cells.

## 3. Discussion

Worldwide, cancer is second only to cardiovascular illnesses as the main cause of death [25,28]. New effective medicines and treatment methods are desperately needed since the prevalence of cancer is steadily rising worldwide. The use of natural substances like hesperidin (Hes) that are safe and have potent anticancer effects might lead to new developments in the treatment of cancer [29]. Consequently, many antitumor mechanisms of this appealing dietary bioflavonoid, such as its proapoptotic, anti-angiogenic, anti-inflammatory, anti-proliferative, anti-invasive, and anti-metastatic properties, have been investigated [30,31,32,33,34]. 

Hes is a significant and commercially accessible flavonoid; however, because of its incredibly poor water solubility, its use has been restricted in many industries. According to reports, flavonoids’ changes in solubility may have an impact on their biological activities [35]. Consequently, chitosan (Cs) has been recognized as a useful tool for therapeutic application due to its adaptable attributes such as expansion and degradability, which aid in managing medicine release rates [36]. Therefore, this study aimed to compare the antioxidant and antitumor activities of Cs, Hes, and Hes-Nanoparticles. 

An experiment with a factorial element provides the chance to investigate the impact that various variables may have on response, making it possible to explore the interactions between elements in an experiment by altering the levels of all the factors simultaneously, rather than one at a time. Two factors, chitosan’s molecular weight (low and intermediate) and temperature (2 and 25 °C), were investigated. Two-level full factorial designs provide guidance for further research because they can detect significant trends. The current study revealed the significant effect of temperature on particle size (the lower the temperature, the smaller the particle size); however, lower temperature had a non-significant effect on zeta potential [37]. A non-significant effect of chitosan’s molecular weight was observed on both particle size and zeta potential. 

The IG technique has many benefits, such as the bioavailability of medicinal formulations, which are significantly boosted by encapsulation. Nearly 100% encapsulation efficiency is possible when the interactions between the polymer and the drug are ideal [38,39,40]. The formulation has muco-adhesion, as well as other essential biological properties, because natural and biodegradable biocompatible polymers are used.

Particles between 200 and 300 nm are thought to be suitable for bypassing RES, glomerular filtration, and biological barriers [41]. Established by the light scattering brought on by the particles’ Brownian motion, dynamic light scattering (DLS) determines particle size [42]. In drug delivery systems utilizing nanoparticles, the system size has an impact on the interaction with tissues and specific cell structures, as well as on pharmacokinetics and clearance [43]. 

When Cs nanoparticles were examined under TEM or SEM, they were often spherical. Moreover, sizes in dry-state conditions (TEM) were lower than those of hydrodynamic size (DLS) [44]. The lower magnification of SEM illustrates cross-linked particles.

The DSC thermogram of the Hes-Nanoparticles revealed that the endothermic melting peak of Hes was absent, showing that Hes was completely encased in amorphous nanoparticles that had the distinct thermal characteristics of pure Hes [45]. There were certain chemical reactions between hesperidin and chitosan (maybe via the formation of hydrogen bonds). This finding can be explained by the disappearance of the characteristic exothermic peak (polymer thermal degradation) of chitosan in the thermogram of Hes-Nanoparticles; on the other hand, the exothermic peak became plateau-like in the drug-free nanoparticle thermogram.

Considering the FTIR spectra, the constant peak observed in Hes-Nanoparticles between 420.74 cm^−1^ and 466.70 cm^−1^ is caused by the out-of-plane and in-plane deformation of rings of Hes that have disappeared, confirming the encapsulation of Hes [24]. The characteristic peak of the hydroxyl group stretching vibration became broader, which may be due to the formation of hydrogen bonding with the Cs amino group [24]. This expected chemical reaction between the Cs amino group and Hes hydroxyl group was consistent with the DSC results. The FTIR spectra of drug-free nanoparticles and chitosan are identical to Cs with a drug ratio of 1.5:1 (*W*/*W*). 

The XRD of Nano-Hes showed the entire disappearance of the distinctive sharp peaks at 2 theta of Hes with a crystalline structure. In the Nano-Hes pattern, these peaks were diminished, suggesting their amorphous nature [46].

The dissolution results were consistent with the swelling test, showing enhanced solubility in 0.1 N HCl, and reaching the complete dissolution of Nano-Hes after 90 min. This can be explained by the solubility characteristic of Cs in an acidic medium (pKa = 6.5) [47].

Moreover, this enhanced solubility can be attributed to the increased surface area according to the New Whitney equation and the ability of hydrogen bond formation [48].

Furthermore, the outcomes of this study show that Hes-Nanoparticles had greater antioxidant activity than Hes and Cs alone. On top of that, it is shown that Nano-Hes outperformed Cs and Hes alone in early cell cycle arrest, cancer cell viability suppression, and enhanced cell apoptosis.

The rise in the solubility and dissolution rates of Hes-Nanoparticles may be the cause of their increased antioxidant activity. Nano-sized medicines preferentially enter tumor tissue via permeable tumor capillaries, and are then maintained in the tumor bed due to decreased lymphatic outflow. “The enhanced permeability and retention (EPR) effect” is the term used to describe this phenomenon [49].

Crucially, reactive radicals can obtain electrons from antioxidants, which changes them into more stable and non-reactive species [50]. As an example, the antioxidant activity of flavonoids, such as Hes, refers to their capacity to transfer an electron or a hydrogen atom, as well as the potential for interactions with other antioxidants [51].

In the same context, the results indicated that the nanoformulation of Hes-Nanoparticles might have improved their possible antioxidant activity. Therefore, nano-formulations may change many physical characteristics, boosting antioxidants’ efficacy [52]. According to earlier research, Hes directly contributes to the scavenging of reactive oxygen species (ROS) through the inhibition of oxidases, reducing α-tocopheryl radicals, metal-chelating action, and the activation of antioxidant enzymes. Hes can also lower superoxide ions in vitro [53,54]. 

Simultaneously, a unique approach to cancer treatment through the use of antioxidants has garnered significant interest recently. Strong antioxidant properties have been discovered in flavonoids, such as hesperidin. Hesperidin has demonstrated antiproliferative and anticancer effects in human cancer cells [55]. Hesperidin and luteolin’s anticancer properties were examined by [56], using human breast cancer cell lines (MCF-7). The findings displayed that apoptosis was triggered by both intrinsic and extrinsic mechanisms, that anti-apoptotic Bcl2 was downregulated, and that pro-apoptotic Bax was upregulated. The cells’ viability was also reduced in a way that depended on both time and dosage. Furthermore, in MCF-7 cells, hesperidin and luteolin markedly decreased miR-21 while raising miR-16 and −34a levels. Accordingly, the study found that luteolin and hesperidin had a promising effect on breast cancer cell lines [57]. The preventive effects of hesperidin on lung cancer invasion, apoptosis, and proliferation have been looked into in different research studies [50]. Hesperidin was applied in varying quantities to treat NCl-H460 and A549 cells. The outcome demonstrated a dose-dependent, substantial reduction in NCl-H460 and A549 cell invasion and activity. Additionally, hesperidin triggered cell death, elevated p53 expressions, and blocked the interaction between p53 and MDMX [58]. The results indicated that hesperidin might be a possible option for the management of different kinds of cancer.

It has been demonstrated that hesperidin induces both internal and extrinsic pathways, which in turn stimulate apoptotic cell death in several cancer cells [59]. The current approach has shown that adding hesperetin to chitosan nanoparticles improves hesperetin binding and internalization, which increases the killing of cancer cells. Chemotherapeutic medicines, resulting in the effective internalization of nanoparticles into lysosomes by cancer cells, have been demonstrated, leading to an increase in cancer cell death [24]. 

## 4. Materials and Methods

### 4.1. Materials

Low-molecular-weight chitosan (50–190 KD), deacetylated degree 90%, was purchased from Sisco Research Laboratories Pvt. Ltd. (SRL), Mumbai, India. Intermediate-molecular-weight chitosan (100–300 KD), deacetylated degree 93–95%, was obtained from Lanxess Company, Thane, India. Sodium tripolyphosphate (85% pure, STPP) was bought from Lanxess Company, India, and acetic acid (purity of 96%) was produced by Research-Lab Fine Chem Industries, Mumbai, India. Also, deionized water was bought from Stakpure, Waters, Milford, MA, USA. High analytical grades were possessed by all other reagents.

### 4.2. Methods

#### 4.2.1. Optimization of Hesperidin Nanoparticles (Hes-Nanoparticles)

A modified ionic gelation technique was used to create chitosan–tripolyphosphate nanoparticles [60]. Chitosan was dissolved at 3% (*w*/*v*) in acetic acid with the aid of a magnetic stirrer (Stuart, Calibre Scientific USA) for 25 min (300 rpm at 50 °C); then, Hes was dispersed into chitosan solution and stirring was continued for an additional one hour. The pH was adjusted to 5 with the aid of 10% sodium hydroxide. Sodium tripolyphosphate (STPP) was dissolved in deionized water to prepare a 1% solution, and then its pH was adjusted with the aid of NaOH (4% *w*/*v*) to 5. Some experiments were performed at a lower temperature. The STPP solution was incubated in an ice bath on a magnetic stirrer (300 rpm); the temperature of the solution was stable at 2 °C. The chitosan/Hes mixture was added dropwise to the previous solution with the aid of a 20 mL syringe (1.8G X1.1/2) with continuous stirring. The stirring was continued for an additional one hour (300 rpm), and the ratio between chitosan, Hes, and NaTPP was 1.5:1:0.5 W/W, respectively. The solution was homogenized for one hour at a speed of 20,000 RPM (IKA, T23, Digital, Ultrax, Staufen, Germany). Ultracentrifugation of the resulting mixture was conducted via a cooling centrifuge for ten minutes at −4 °C at 10,000 rpm (Centurion Scientific, Chichester, UK). The nanoparticles were washed with deionized water twice and then collected for further freeze-drying (Christ Benchtop Freeze dryer, Osterode am Harz, Germany). The supernatant and wash solution was mixed for the determination of entrapment efficiency (EE). Chitosan cross-linked with STPP as control nanoparticles was prepared with the same procedure, except for the addition of Hes powder.

#### 4.2.2. Characterization of Hes-Nanoparticles 

##### Swelling Test

Pre-weighed Hes-Nanoparticle powder was submerged in an excessive swelling medium (DW, 0.1 N HCl, buffer pH 4.6 and 6.8) at 37 °C. After blotting the excess solution off the surface with the aid of filter paper, the hydrogel was taken out of the solution at different intervals (0.5, 1, 3, and 4) and weighed. A swelling test in 0.1 N HCL was conducted at 37 °C for 72 h to compare the swelling behavior between HES and Hes-Nanoparticle powder. After the filtration of the filter, the residues were weighed again. The mean values of measurements made in triplicate were calculated according to the following equations to determine the swelling ratio.

(3) $$\mathrm{Swelling}\;\mathrm{ratio}=\;\frac{W_{s}-W_{d}}{W_{d}}$$

(4) $$\mathrm{Volume}\;\mathrm{swelling}\;\mathrm{factor}=\;\;\frac{V_{t}}{V_{0}}$$

(5) $$\mathrm{Sol}\;\mathrm{fraction}=\;\frac{\;W_{i}-W_{d}}{W_{i}}$$

Here, W_d_ is the mass after it has dried, and W_s_ is the mass after it has swelled [61]. In contrast, V_t_ and V_0_ represent the starting and remaining volumes, respectively [62]. The proportionate rise in weight of the hydrogel caused by water absorption is known as the swelling ratio. The sol fraction is the percentage of the polymer that, following a cross-linking process, is not attached to a cross-linked network. Sol fraction declines over time, reflecting polymer loss and indicating the degree of hydrogel deterioration and degradation [63].

##### The Percentage Yield

The percentage yield (%) of the nanoparticles is a key factor since it can be used to predict the likelihood of industrial scale-up and the feasibility of a procedure. The total amount of powder was determined after lyophilization using an analytical weighing scale (Sartorious, Ann Arbor, MI, USA), and the yield % was calculated using the formula below.

(6) $$\mathrm{The}\mathrm{percentage}\mathrm{yield}=\frac{Total\;amount\;of\;HNP}{Total\;amount\;of\;all\;ingredients\;(STPP+chitosan+drug)}\times 100$$

##### Drug Entrapment Efficiency (DEE)

Both the loading capacity (LC) and drug entrapment efficiency (DEE) were computed using the indirect technique. After the centrifuged supernatant and wash were collected, the amount of entrapped drug was calculated using a calibration curve. The complete analysis was carried out in three duplicates to minimize handling errors. While DEE is the proportion of the medication that is successfully encapsulated inside the system from the entire drug that was initially added, LC is the percentage of the drug that is successfully loaded on the specific mass of nanoparticles. DEE and LC can be calculated according to the following equations.

(7) %DEE=(Totaldrugconc.−Supernatantdrugconc./Totaldrugconc.×100)

(8) $$\%\mathrm{LC}=\frac{\mathrm{Total}\mathrm{amount}\mathrm{of}\mathrm{drug}\mathrm{added}-\mathrm{Amount}\mathrm{of}\mathrm{untrapped}\mathrm{drug}}{\mathrm{Total}\mathrm{mass}\mathrm{of}\mathrm{HNPs}}\times 100$$

##### Average Particle Size and Zeta Potential Evaluation

The effectuality of nanoparticles is dependent on their particle size; on the other hand, zeta potential is a marker of colloidal stability [64]. The Zeta Sizer Nano (Malvern Panalytical Ltd., Enigma Business Park, Malvern, UK) was used to assess particle size and zeta potential.

##### Scanning Electron Microscopy (SEM)

SEM analyses were performed to evaluate the shape and surface characteristics of Hes, Hes-Nanoparticles, and drug-free nanoparticles. After being dissolved in alcohol using a sonicator, Hes and the lyophilized Nano-Hes were distributed over a glass slide, allowed to dry completely, and then transferred to the top of a metal cup on a silicon electro-conductive chip. Using a 10 kV electron acceleration voltage field-emission scanning electron microscope (JEOL, JSM-6510LV, Tokyo, Japan) at various magnifications, the materials were coated with gold for one minute on their stubs.

##### Transmission Electron Microscopy (TEM)

The nanoparticles were suspended in ethyl alcohol; after that, the samples were mounted on a carbon grid and dried. The samples were observed and photographed with a transmission electron microscope (TEM, JEM2100F electron microscope, JEOL, Ltd., Tokyo, Japan).

##### Thermal Stability (DSC)

Utilizing TA devices from Waters LIC in the USA, the thermal behavior of the raw material samples was investigated using differential scanning calorimetry (chitosan, NTPP, and Hes), considering the drug-free nanoparticle samples and the Hes-Nanoparticles. A microbalance (Sartorius, Göttingen, Germany) was used to precisely weigh each sample (3–4 mg), and the samples were heated from 50 to 350 °C at a rate of 10 °C/min. 

##### X-ray Diffraction Analysis (XRD)

Chitosan, Hes, and Hes-Nanoparticles underwent XRD analysis. The X-ray diffractograms based on Bragg’s law were acquired using an XRD diffractometer (APD2000 pro, GNR, Italy; software CRYSTAL IMPACT (2003–2014), Bonn, Germany) with CuK radiation, 35 kV of monochromatic voltage, and a 25 mA electric current. The range of the 2 θ diffraction angle was 4.95° to 79.75°.

##### Fourier-Transform Infrared Spectroscopy (FTIR) Analysis

The FTIR analysis, which was performed to evaluate the interactions and compatibility of the formulation’s components, emphasized the stability of the suggested system [65]. Hes and chitosan powder in addition to Hes-Nanoparticles and drug-free nanoparticles were analyzed using BRUKER (Billerica, MA, USA). 

##### In Vitro Drug-Release Study

Comparative dissolution patterns in different pHs (4.6 and 6.8) were identified to compare the dissolving behaviors of Hes and Hes-Nanoparticles. The volume of dissolution media was 100 mL, and the speed of rotation was 75 rpm at 37 °C (Stuart, Calibre Scientific, Holland, MI, USA). Precisely weighted samples with an equivalent of 10 mg of Hes were transferred into a dialysis membrane sac (dialysis tubing 29.6 × 45 mm, (FREY Scientific, Nashua, NH, USA) and dropped onto the dissolving media. Samples of the dissolution medium (5 mL) were collected at numerous time interludes (0.5, 1, 2, 3, 4, and 24 h). Fresh medium (5 mL) was used to make up for samples that were withdrawn.

Dissolution in 0.1 N HCl (900 mL) was performed using USP type II equipment (Copley Scientific, Nottingham, UK). The paddles revolved at a speed of 75 rpm, while the temperature was kept at 37 °C. Accurately weighted samples equivalent to 10 mg of Hes were added to the dissolving media. Samples of the dissolution medium (3 mL) were filtered via a 0.2 mm syringe filter and examined spectrophotometrically (Shimadzu RF-6000, Kyoto, Japan) at 238 nm. A new medium was used to make up for samples that were withdrawn. Three separate dissolving experiments were run, with samples being taken at 10, 20, 40, and 90 min.

#### 4.2.3. Antioxidant Activity Assay

##### DPPH Radical Scavenging Assay

The DPPH radical scavenging experiment was carried out for chitosan, Hes, and Hes-Nanoparticles at different concentrations (3.9, 7.8, 15.62, 31.25, 62.5, 125, 250, 500, and 1000 μg/mL) using the method described in [66]. The absorbance at 517 nm was measured using a UV/visible spectrophotometer (UV-VIS Milton Roy). Ascorbic acid was used as a reference chemical. The IC_50_ value was obtained by using the Log dose inhibition curve (*n* = 3). The percentage of DPPH scavenging effect was determined using the following formula:

DPPH scavenging effect (%) or percent inhibition = A0 − A1/A0 × 100 (9)

where A0 represents the absorbance of the control response and A1 represents the absorbance in the presence of a test or standard sample.

##### ABTS Radical Scavenging Assay

ABTS radical scavenging activity was identified for chitosan, Hes, and Hes-Nanoparticles at different concentrations (3.9, 7.8, 15.62, 31.25, 62.5, 125, 250, 500, and 1000 μg/mL), following [67] with minor adjustments. A spectrophotometer was used to measure the absorbance at 734 nm following a 6 min incubation period. The following formula was employed to assess antioxidant activity:

%Inhibition= (A control − A sample)/A control × 100(10)

A control = Absorption of the negative control with solution preparation.

A sample = Sample absorbance after six minutes.

Gallic acid was used as a reference chemical. A graph showing the concentration of the sample required to scavenge 50% of the ABTS free radicals (*n* = 3) was used to obtain the IC_50_ value.

#### 4.2.4. Antitumor Activity

Viability test for chitosan, hes, and hes-nanoparticles demonstrated anticancer efficacy against MDA-MB-231 (breast cancer cell line).

The breast cancer cell line (MDA-MB-231) was acquired from the National Cancer Institute in Cairo, Egypt. The tumor cells were cultured in a 37 °C humid environment with 5% carbon dioxide using Corning 96-well tissue culture plates. The cells were suspended in the medium at a concentration of 5 × 104 cells/well. Next, after 48 h of exponential development, the cells were treated with chitosan, Hes, or Hes-Nanoparticles at doses of 0, 25, 50, 75, 100, and 125 (µg mL^−1^, 48 h). Subsequently, each well was supplemented with 10 µL of the 12 mM MTT stock solution (Vybrant^®^ MTT Cell Proliferation Assay Kit, V-13154) and incubated for 4 h at 37 °C. Following a thorough mixing with the pipette, 50 µL of DMSO was added to each well, and the mixture was incubated for 10 min at 37 °C. Utilizing a microplate reader (ELx 800, Bio-Tek Instruments Inc., Santa Clara, CA, USA) at 540 nm, the absorbance was measured [68]. The optical densities of the treated cells (A) and the untreated cells (B) are represented by the following formula:

Rate of inhibition (%): (A/B) × 100 (11)

Furthermore, the IC_50_ was computed using the GraphPad Prism 10.2.3 program (San Diego, CA, USA).

##### Cell-Cycle Analysis

After treatment with chitosan, Hes, or Hes-Nanoparticles, the MDA-MB-231 breast cancer cell line’s cell-cycle distribution was investigated by utilizing flow cytometric analysis of the IC_50_ values discovered by the MTT test. Following the stimulation of the cells with Hes, Nano-Hes, or chitosan, the culture media were carefully removed, PBS was added, and the mixture was gently agitated before the PBS was taken out. One milliliter of trypsin was added, vigorously mixed, and allowed to digest in the incubator. The trypsin digestion process was finished by removing the cells from the incubator and placing them in a 3 mL serum-containing medium. Using a pipette, the cells were resuspended and put into the centrifuge tube. The supernatant was then extracted using centrifugation at 1000 rpm for five minutes at room temperature [69]. Following this, three milliliter PBS resuspension cells were added. After 75% alcohol was used to revive the cells, they were chilled at 4 °C for the duration of the night. Then, we collected the supernatant after centrifuging at 1000 rpm for five minutes at room temperature. Following three PBS washes, the cell cycle was assessed using flow cytometry (BD AccuriTM C6 Plus Flow Cytometer). A propidium iodide staining solution was then added, and the cells were stained for thirty minutes at 37 °C [69]. BD Biosciences’ AccuriTM C6 software was used to calculate the percentage of cells in each cell-cycle phase.

##### Annexin-V/PI Double-Staining Assay

A well plate was injected with cells that were in the exponential growth phase. Following the cell-collection process, the culture media were slurped into the centrifuge tube, the cells were cleaned with 1 mL of 1× Binding Buffer, and the cell pellet was resuspended in 100 μL of 1× Binding Buffer. The cells were then centrifuged at 300× *g* for 10 min. Subsequently, every 106 cells were treated with 10 μL of annexin-V tagged with a fluorescent dye (annexin V-FITC), which was carefully mixed and incubated for 15 min at room temperature without light. Then, 500 μL of 1× Binding Buffer was added to each 106 cells for a second round of washing. The cells were then centrifuged at 300× *g* for ten minutes, and the cell pellet was resuspended in 500 μL of 1× Binding Buffer. Lastly, 5 μL of propidium iodide solution was added right before the BD AccuriTM C6 Plus Flow Cytometer was used for analysis [70]. 

### 4.3. Statistical Analysis 

Every experiment was carried out at least three times. Mean ± SD is used to express all data.

## 5. Conclusions

Finally, nanotechnology is greatly improving the efficient delivery of bioactive materials. In the realm of food medicine, using nanoscience has already been shown to be a revolutionary technical breakthrough. Hesperidin-loaded nanoparticles may be able to improve some of the therapeutic uses of pure hesperidin, such as the management of cardiovascular, viral, and respiratory disorders, and cancer.

## Figures and Tables

**Table 1:** Full factorial design.

Factors	2	Base design	2, 4
Run	12	Replicates	3
Block	1	Center pts(total)	0

**Table 2:** The results of average particle size (nm) and zeta potential (mV) in response to full factorial design experiment (*n* = 4).

Formulation Code	Independent Variables		Response	
	Cs Molecular Weight (KD)	Temperature °C	Particle Size (nm) Mean ± SD	Zeta Potential (mV) Mean ± SD
F1	100	2	112.4 ± 63.78	31.33 ± 9.71
F2	300	25	423.2 ± 63.40	20.32 ± 3.67
F3	100	25	278.6 ± 168.25	23.75 ± 0.66
F4	300	2	168.1 ± 135.89	30.04 ± 10.04

**Table 3:** Swelling ratio, sol fraction, and volume swelling factor (VSF) of Hes-Nanoparticles in different media.

	**Swelling Ratio**				**VSF**
	**0.5 h**	**1 h**	**3 h**	**4 h**	**4 h**
Water	15.7 ± 6.99	26.5 ± 11.66	23.1 ± 9.25	17.6 ± 6.79	0.5
pH 4.6	26.5 ± 11.23	29.7 ± 12.23	22.97 ± 8.47	19.1 ± 6.61	0.5
pH 6.8	27.1 ± 11.41	30.1 ± 12.38	24.0 ± 9.05	19.5 ± 6.57	0.5
0.1 N HCl	25.9 ± 10.92	37.7 ± 15.58	24.0 ± 8.99	24.1 ± 8.55	0.42
	**Sol fraction**				
	**0.5 h**	**1 h**	**3 h**	**4 h**	
Water	0.94 ± 0.009	0.96 ± 0.005	0.96 ± 0.006	0.95 ± 0.013	
pH 4.6	0.96 ± 0.004	0.97 ± 0.002	0.96 ± 0.003	0.95 ± 0.008	
pH 6.8	0.96 ± 0.003	0.97 ± 0.002	0.96 ± 0.005	0.95 ± 0.004	
0.1 N HCl	0.96 ± 0.004	0.97 ± 0.001	0.96 ± 0.004	0.96 ± 0.004	

**Table 4:** Polymeric Hes-Nanoparticle characterization *.

Parameter	Mean	Range
% Yield	91.51 ± 2.7	88.66 to 94.0
% EE	85.92 ± 1.9	84.28 to 87.93
% LC	30.69 ± 0.66	30.1 to 31.4
Particle size (nm)	184.1 ± 20.03	161 to 196.7
Zeta potential (mV)	−29.07 ± 9.78	−20.61 to −42.75
PDI	0.233 ± 0.061	0.172 to 0.295

* Mean ± SD, (*n* = 3).

**Table 5:** The activity of chitosan, Hes, and Hes-Nanoparticles against breast cancer cell line (MDA-MB-231, incubation for 48 h) with IC_50_ = 116.63 ± 2.96 μg/mL, IC_50_ = 110.82 ± 5.89 μg/mL and IC_50_ = 110.82 ± 5.89 μg/mL, respectively *.

**Chitosan Concentration** **(µg/mL)**	**Viability (%)**	**Inhibition (%)**
0	100	0
25	87. 44 ± 3.9	12.65 ± 1.22
50	73.11 ± 9.13	26.88 ± 1.15
75	65.13 ± 2.6	34.87 ± 2.35
100	55.43 ± 3.66	44.57 ± 2.38
125	53.17 ± 1.08	49.17 ± 3.05
**Hes Concentration** **(µg/mL)**	**Viability (%)**	**Inhibition (%)**
0	100	0
25	73.53 ± 1.75	26.47 ± 1.6
50	65.74 ± 3.35	34.26 ± 2.24
75	60.65 ± 3.95	39.35 ± 1.42
100	52.62 ± 5.99	47.38 ± 2.52
125	47.24 ± 1.07	53.76 ± 1.93
**Hes-Nanoparticle Concentration** **(µg/mL)**	**Viability (%)**	**Inhibition (%)**
0	100	0
25	66.69 ± 5.42	33.3 ± 2.4
50	60.31 ± 9.05	39.69 ± 1.22
75	56.66 ± 1.71	43.34 ± 3.40
100	48.62 ± 0.7	51.38 ± 1.52
125	36.54 ± 2.5	63.46 ± 2.8

* Data are expressed as mean ±SD, *n* = 3. Hes: Hesperidin and Hes-Nanoparticles: chitosan/hesperidin nanoparticles.

## Data Availability

Data are contained within the article.
